# Combined effects of a third-generation bisphosphonate, zoledronic acid with other anticancer agents against murine osteosarcoma

**DOI:** 10.1038/sj.bjc.6603548

**Published:** 2007-01-23

**Authors:** N Horie, H Murata, S Kimura, H Takeshita, T Sakabe, T Matsui, T Maekawa, T Kubo, S Fushiki

**Affiliations:** 1 Department of Orthopaedics, Graduate School of Medical Science, Kyoto Prefectural University of Medicine, 465 Kawaramachi-Hirokoji, Kamigyo-ku, Kyoto 602-8566, Japan; 2 Department of Pathology and Applied Neurobiology, Graduate School of Medical Science, Kyoto Prefectural University of Medicine, 465 Kawaramachi-Hirokoji, Kamigyo-ku, Kyoto 602-8566, Japan; 3 Department of Transfusion Medicine and Cell Therapy, Kyoto University Hospital, 54 Kawahara-cho shogoin, Sakyo-ku, Kyoto 606-8507, Japan

**Keywords:** osteosarcoma, bisphosphonates, zoledronic acid, combined effects

## Abstract

Bisphosphonates (BPs) are widely used to treat bone diseases and also appear to possess direct antitumour activity. We have previously reported that third-generation BPs such as zoledronic acid (ZOL) and minodronic acid (YM529) synergistically augment the effects of anticancer agents in various cancer cells. Recently, we have also reported the antitumour effects of YM529 on murine osteosarcoma cells. As YM529 has not been clinically available, we herein focused on the anti-osteosarcoma effects of ZOL which is clinically available. In addition to ZOL alone, we evaluated the concurrent or sequential combined effects of ZOL with other anticancer agents against murine osteosarcoma cell lines. ZOL showed almost same anti-osteosarcoma activity compared with YM529 and more sensitive growth inhibitory effects against osteosarcoma cells than normal cells. Moreover, ZOL acted synergistically *in vitro* when administered concurrently with paclitaxel (PAC) or gemcitabine (GEM), not only in wild-type osteosarcoma cells but also in P-glycoprotein (P-gp)-overexpressing osteosarcoma cells, which were much less sensitive against each anticancer agent. Furthermore, 24 h of ZOL pretreatment significantly augmented the sensitivity of doxorubicin (DOX), PAC or GEM against osteosarcoma cells. These findings suggest that combined administration of ZOL with other anticancer agents may improve the osteosarcoma treatment.

Osteosarcoma is a high-grade malignant bone neoplasm that occurs in children and adolescents. Recently, the prognosis of these patients has improved substantially owing to the development of various adjuvant chemotherapies. However, these chemotherapies are not fully effective and as a result, 20% of all osteosarcoma patients still die owing to tumour metastasis (Link, 1993; Unni, 1996; Bacci *et al*, 2006). As a consequence, various new osteosarcoma therapies have been investigated worldwide, with many clinical trials performed on novel agents.

Bisphosphonates (BPs) are widely used to treat bone diseases such as osteoporosis, which is caused by excessive bone resorption or metastatic bone involvement (Fleisch, 2002). We have previously reported that third-generation BPs such as zoledronic acid (ZOL) and minodronic acid YM529 show direct antitumour effects and synergistically augments the effects of anticancer agents in various cancer cell lines (Kuroda *et al*, 2003; Kimura *et al*, 2004; Matsumoto *et al*, 2005; Segawa *et al*, 2005; Yuasa *et al*, 2005). Recently, several investigators have reported the anti-osteosarcoma effects of third-generation BPs *in vitro* (Evdokiou *et al*, 2003; Kubista *et al*, 2006; Kubo *et al*, 2006; Tenta *et al*, 2006) and *in vivo* (Heymann *et al*, 2005; Ory *et al*, 2005). We have also reported that YM175 and YM529 inhibit the growth of murine osteosarcoma cell lines in a time- and dose-dependent manner by preventing prenylation of small GTPases and might be subject to multi-drug resistance mechanism in osteosarcoma cells (Horie *et al*, 2006).

There are numerous reports concerning the combined effects of third-generation BPs with anticancer agents in various cancer cell lines. However, only Heymann *et al* (2005) reported the combined effect of ZOL with ifosfamide in osteosarcoma cell lines. As YM529 has not been clinically available, we herein focused on the anti-osteosarcoma effects of ZOL which is clinically available. In addition to ZOL alone, we herein investigated the concurrent or sequential combined effects of ZOL against two murine osteosarcoma cell lines not only with commonly used agents for osteosarcoma such as doxorubicin (adriamycin, DOX), cisplatin (CDDP) and methotrexate (MTX) (Bacci *et al*, 2006) but also with novel agents such as imatinib mesylate (IM), paclitaxel (PAC) and gemcitabine (GEM) which have recently been analysed for osteosarcoma treatment (Verweij *et al*, 2000; McGary *et al*, 2002; Okuno *et al*, 2002).

## MATERIALS AND METHODS

### Reagents

ZOL (1-hydroxy-2-(1H-imidazole-1-yl) ethylidene-bisphosphonic acid) and IM were obtained from Novartis Pharma AG (Basel, Switzerland). DOX (from Kyowa Hakko Kogyo Co. Ltd., Tokyo, Japan), CDDP (from Nihon Kayaku Co. Ltd., Kyoto, Japan), PAC (from Bristol–Myers Squibb, New York, USA) and GEM (from Eli Lilly KK, Kyoto, Japan) were provided by each company MTX, verapamil and ethylenediaminetetracetic acid (EDTA) were purchased from Sigma Aldrich (Tokyo, Japan). ZOL, DOX, IM and PAC were dissolved in Ca^−^ Mg^−^ phosphate-buffered saline (PBS). MTX was dissolved in 0.1 N NaOH and then further diluted in PBS. All diluted solutions were stored at −20°C. Appropriate drug concentrations were made by dilution with fresh medium immediately before each experiment.

### Cell lines

MOS cell line was established from the murine osteosarcoma model developed at Massachusetts General Hospital (Choi *et al*, 1979). P-glycoprotein (P-gp)-overexpressing cell line which was established by stepwise increments of DOX, MOS/ADR was generated as previously reported (Takeshita *et al*, 1996). Murine osteosarcoma cell line, LM8 was established from the murine Dunn osteosarcoma cell line (Asai *et al*, 1998). Normal human dermal fibroblasts (NHDF) cell line was purchased from Kurabo (Osaka, Japan). These cells were maintained in Dulbecco's modified Eagle’s medium supplemented with 15 mM HEPES buffer, 10% foetal bovine seruman antibiotic solution of penicillin (100 U ml^−1^) and streptomycin (100 *μ*g ml^−1^). Normal murine osteoblast cells were isolated from murine skull bone as described elsewhere (Takahashi *et al*, 1988). These cells were maintained in α-MEM supplemented with 10% foetal bovine serum and an antibiotic solution of penicillin (100 U ml^−1^) and streptomycin (100 *μ*g ml^−1^). All cells were cultured at 37°C in a fully humidified incubator with 5% CO_2_. All experiments described were performed at least three times using cells in the exponential growth phase.

### Concurrent exposure to ZOL and other anticancer agents

Proliferation of the cell lines was determined using the methyl-thiazol-diphenyl-tetrazolium (MTT) assay, as previously described (Hansen *et al*, 1989). MOS, MOS/ADR or LM8 and osteoblast or NHDF cells were cultivated in a flat-bottomed 96-well plate (Greiner Labortechnik, Frickenhausen, Germany) at 5 × 10^3^ and 1 × 10^4^ cells per well, respectively in 100 *μ*l of medium and incubated with various concentrations of ZOL alone or in combination with other anticancer agents such as DOX, CDDP, MTX, IM, PAC or GEM for 48 h. The means of six data values for each treatment were calculated. For all the cell lines, we evaluated a linear relationship between the degree of proliferation and cell number within the range of the experiment. Half-maximal inhibition constants (IC_50_) were determined using the nonlinear regression programme CalcuSyn (Biosoft, Cambridge, UK). To investigate the effect of combining ZOL with other anticancer agents, the MOS or LM8 cells were treated with six concentrations (0.25, 0.5, 0.75, 1.0, 1.5 or 2.0 × IC_50_) of ZOL alone and ZOL combined with another anticancer agent. The fraction affected (Fa) (i.e. Fa of 0.25 is equivalent to 75% viable cells) and the combination index (CI) were calculated with CalcuSyn (Chow *et al*, 2000). This method enables quantification of synergism (CI<1) and antagonism (CI>1) at different dose and effect levels. Combination index calculations were made under the assumption that the mechanisms of drug action were not mutually exclusive.

### Sequential exposure of cells to ZOL and other anticancer agents

We next investigated the effect of a sequential exposure regime with ZOL, followed by the other anticancer agents. MOS or LM8 cells were incubated in 96-well plates at a density of 1.5 × 10^3^ in 100 *μ*l of medium per well for 24 h, then incubated with 1.0 *μ*M ZOL for MOS cells or 10 *μ*M ZOL for LM8 cells for 24 h. After the osteosarcoma cells were washed thrice in PBS, the second anticancer agent was added to the respective wells. After a further 48 h, the rate of growth inhibition was evaluated by MTT assay. Data from three independent experiments were collected and the Student’s *t*-test was used to evaluate the efficacy of sequential treatment of ZOL and other agents and to compare the effects of each anticancer agent alone. *P*-values of less than 0.05 were considered statistically significant and were derived from two-sided statistical tests.

### Cell cycle analysis

To explore the possible mechanisms of combined effects of ZOL and other agents, MOS or LM8 cells were analysed for cell cycle alterations by staining with propidium iodide (Sigma Aldrich) after exposure to ZOL and/or anticancer agents for 24 h, as previously described (Kimura *et al*, 1995). The stained nuclei were analysed using a FACSCalibur flow cytometry (Becton Dickinson, Japan). DNA histograms were created using Cell Quest™ software for Apple Macintosh (Becton Dickinson).

## RESULTS

### Growth inhibitory effects of ZOL against murine osteosarcoma cells

**Figure 1:**
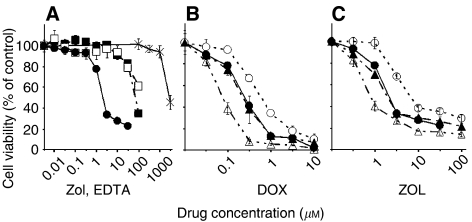
Effect of ZOL on growth of cells. (**A**) The ability of ZOL to inhibit the growth of the murine osteosarcoma MOS cells (•), murine osteoblast cells (▪) and human fibroblast cell line (□) was determined by MTT assay. And also, that of EDTA to inhibit the growth of MOS cells (×) was determined. The ability of DOX (**B**) and ZOL (**C**) to inhibit the growth of the P-gp-overexpressing MOS/ADR cell line (○) and its parental MOS cell line (•) was determined by MTT assay. MOS/ADR cell line was 4.4 times more resistant to DOX than MOS cells and was also not as sensitive to ZOL. When cells were incubated with 1 *μ*M of verapamil (▴), the ability of DOX and ZOL to inhibit the growth of the MOS/ADR cell line became as sensitive as its parental MOS cell line. Furthermore, 10 *μ*M of verapamil (□) strengthened the inhibitory effect of these agents on the MOS/ADR cell line more than on the MOS cell line.

ZOL inhibited the growth of murine osteosarcoma cells dose dependently, whereas in normal cells such as murine osteoblast cells and human fibroblast cells, NHDF were much less sensitive to ZOL. Growth of MOS cells was not inhibited by up to 1000 *μ*M EDTA (Figure 1A). The IC_50_ values of ZOL for MOS, LM8, osteoblast and NHDF cells after 48 h exposure were 1.56, 7.36, 72.4 and 145.3 *μ*M, respectively. ZOL showed almost same anti-osteosarcoma activity compared with YM529 (Table 1).

P-gp-overexpressing MOS/ADR cell line was 4.4 times more resistant to DOX than its parental MOS cell line (Table 1, Figure 1B). Similarly, MOS/ADR cells were not as sensitive to ZOL as MOS cells (Figure 1C). The IC_50_ value of ZOL for the MOS/ADR cells after 48 h exposure was 7.10 *μ*M which was 4.6 times more resistant to ZOL than parental MOS cells (Table 1). We next examined the combined effects of a P-gp inhibitor, verapamil with ZOL against P-gp-overexpressing MOS/ADR cells. Verapamil (1 *μ*M) augmented the effects of ZOL on MOS/ADR cells and restored the sensitivity of MOS/ADR cells almost same as of parental MOS cells. Verapamil alone up to 30 *μ*M had no growth-inhibitory effects on these cell lines (data not shown). These results suggested that ZOL may be influenced by P-gp related multi-drug resistance system in osteosarcoma cell lines.

Growth-inhibitory effects of other anticancer agents against osteosarcoma cells are summarised in Table 1.

### Concurrent combined effects of ZOL with other anticancer agents

**Figure 2:**
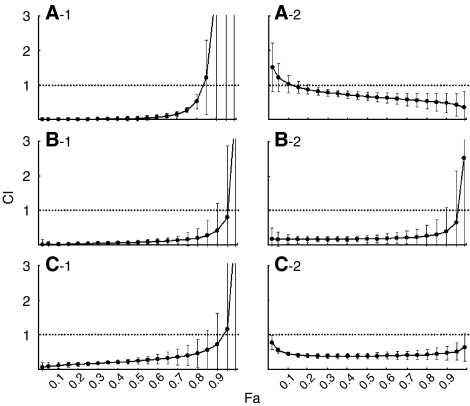
Effect of the concurrent treatment with ZOL and anticancer agents on murine osteosarcoma cell lines growth. The combination index (CI) is plotted as a function of the fraction affected (Fa), which represents the percentage of growth inhibition (e.g. 0.5=50%) and was evaluated using the MTT assay. Combinations of multiple equipotent agent concentrations were analysed for synergistic (CI<1), additive (CI=1), or antagonistic (CI>1) effects. Concurrent exposure to PAC (-1) and GEM (-2) on LM8 cells (**A**), MOS cells (**B**) and MOS/ADR cells (**C**). Data are presented as the mean±s.d. of three independent experiments.

At first, we examined the combined effects of ZOL with commonly used agents for osteosarcoma such as DOX, CDDP and MTX. When combined with CDDP, the CIs at Fa 0.5 and Fa 0.8 were less than 1.0±1 s.d. in both MOS and LM8 cells, except at Fa 0.5 for LM8 cells, indicating that the effects of combination with CDDP were synergistic rather than additive effects. DOX and MTX additively augmented the effects of ZOL (Table 2). Next, we explored the combined effects of ZOL with IM, PAC and GEM, which were expected to be novel agents for osteosarcoma. IM also showed additive effects with ZOL (Table 2). Interestingly, PAC and GEM demonstrated significant synergistic effects with ZOL not only in MOS and LM8, but also in P-gp-overexpressing cell line, MOS/ADR (Table 2, Figure 2).

### Sequential combined effects of ZOL with other anticancer agents

**Figure 3:**
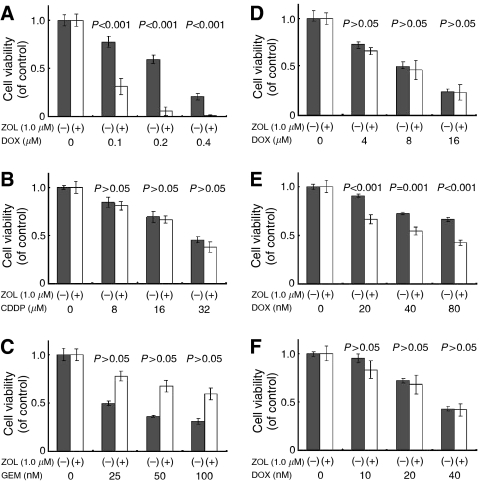
Effect of sequential combinations of ZOL and anticancer agents on MOS cell line growth. MOS cells were pretreated with 1.0 *μ*M ZOL for 24 h, washed thrice in PBS and then treated for 48 h with the second anticancer agents, namely, DOX (**A**), CDDP (**B**), MTX (**C**), IM (**D**), PAC (**E**) or GEM (**F**). Data are presented as the mean±s.d. of three independent experiments.

**Figure 4:**
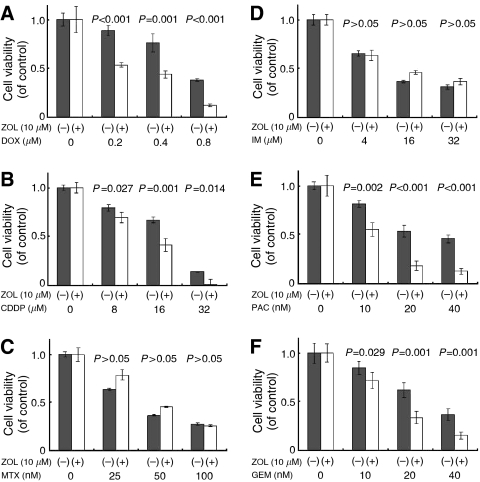
Effect of sequential combinations of ZOL and anticancer agents on LM8 cell line growth. LM8 cells were pretreated with 10 *μ*M ZOL for 24 h, washed thrice in PBS and then treated for 48 h with the second anticancer agents, namely, DOX (**A**), CDDP (**B**), MTX (**C**), IM (**D**), PAC (**E**) or GEM (**F**). Data are presented as the mean±s.d. of three independent experiments.

Cytotoxic effects of DOX on both MOS (Figure 3A) and LM8 cells (Figure 4A) and of CDDP on LM8 cells (Figure 4B) were sinificantly enhanced by a 24 h pretreatment with ZOL (*P*<0.05), whereas the cytotoxic effects of MTX were antagonised by the ZOL pretreatment of both osteosarcoma cell lines (Figures 3C and 4C). Cytotoxic effects of PAC on both MOS (Figure 3E) and LM8 cells (Figure 4E) and of GEM on LM8 cells (Figure 4F) were significantly enhanced by a 24 h pretreatment with ZOL (*P*<0.05), whereas the cytotoxic effects of IM were affected significantly by ZOL pretreatment, neither in MOS (Figure 3D) nor in LM8 cells (Figure 4D).

### Alterations of cell cycle by ZOL, PAC or GEM alone

As described above, the significant synergistic effects of PAC with ZOL was seen in both MOS and LM8 cells, and those of GEM were demonstrated only in LM8 cells. To investigate the possible mechanisms underlying the synergistic interaction between ZOL and PAC or GEM, we analysed the effects of these anticancer agents on cell cycle. Alterations of cell cycle by 24 h exposure to ZOL, PAC or GEM in MOS and LM8 cells are summarised in Table 3. After 24 h exposure to ZOL at 2.0 *μ*M for MOS cells or at 15 *μ*M for LM8 cells, the percentages of cells in the S phase increased without significant increase in the sub-G_1_ phase. After 24 h exposure to PAC at 25 nM for MOS or at 10 nM for LM8 cells, the percentages of cells in the G_1_ and S phases decreased and those in the G_2_/M and sub-G_1_ phase increased. After 24 h exposure to 25 nM GEM, the percentages of cells in the S and G_2_ phases decreased, and those in sub-G_1_ and G_1_ phases increased.

### Combined effects of ZOL with PAC or GEM on the alterations of cell cycle

**Figure 5:**
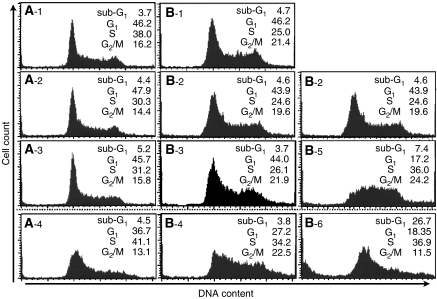
Cell cycle analysis of combined treatment of ZOL with PAC or GEM. The effect of agents on the cell cycle was evaluated by flow cytometry of osteosarcoma cells that had been exposed to different agent concentrations for 24 h. MOS cells (**A**); untreated (−1), 0.5 *μ*M ZOL (−2), 5 nM PAC (−3), 0.5 *μ*M ZOL with 5 nM PAC (−4) and LM8 cells (**B**); untreated (−1), 10 *μ*M ZOL (B-2), 5 nM PAC (−3), 10 *μ*M ZOL with 5 nM PAC (−4), 10 nM GEM (−5) and 10 *μ*M ZOL with 10 nM GEM (−6). Cell cycle distribution (%) of each agent is shown. The data shown are representative of three independent experiments.

When MOS cells were treated with 0.5 *μ*M ZOL combined with 5 nM PAC for 24 h, there was an increase in the proportion of MOS cells in S-phase despite the fact that neither drug at this concentration affected the cell cycle when used individually (Figure 5A-1-4). These results are same as on LM8 cells treated with 10 *μ*M ZOL combined with 5 nM PAC for 24 h (Figure 5B-1-4). These suggested that when ZOL is combined with PAC, PAC might augment the ability of ZOL to produce S-phase arrest. When LM8 cells were treated with 10 *μ*M ZOL combined with 10 nM GEM for 24 h, there was an increase in the ratio of LM8 cells in sub-G_1_, although neither drug concentration produced this effect when applied by itself (Figures 5B-2, 5 and 6). This indicated that when combined with GEM, ZOL might augment the sub-G_1_ effect of GEM on the cell cycle.

## DISCUSSION

Our previous study revealed that when used as single agents, the third-generation BPs, YM175 and YM529, possess antitumour activity in an osteosarcoma cell line *in vitro*, and when these effects were compared with the effect of BPs on other tumour cell lines, YM175 and YM529 produce stronger antitumour effects on the murine osteosarcoma cell line than other cancer cell lines. However, the effects of YM175 and YM529 were impaired against a P-gp-overexpressing osteosarcoma cell line (Horie *et al*, 2006). As YM529 has not been made clinically available yet we investigated the anti-osteosarcoma effects of ZOL, another third-generation BP, which is clinically available and as potent as YM529 at inhibiting bone resorption *in vivo* (Widler *et al*, 2002).

To investigate specificity of ZOL against osteosarcoma cells, we examined the inhibitory effects of ZOL against both osteosarcoma cell lines and normal cells such as murine osteoblast and NHDF cells. Moreover, to test the hypothesis that ZOL could have chelating action, we examined the growth-inhibitory effects of the most commonly used chelating agent EDTA on osteosarcoma cell lines. Lower concentration of ZOL did not inhibit the growth of normal cells, and 1000 *μ*M EDTA did not inhibit the growth of osteosarcoma cells (Figure 1A). If the effect of ZOL depends on its chelating mechanism, divalent cations can inhibit it. However, the antiproliferative effect of ZOL was reported to be strengthened by the addition of divalent cations, while that of EDTA was weakened by the addition of divalent cations (Reinholz *et al*, 2000). These findings suggest that ZOL selectively inhibited the growth of osteosarcoma cells independently by its chelating effects.

The interaction between ZOL and P-gp is still controversial. P-gp-overexpressing MOS/ADR cell line was 4.6 times more resistant to ZOL than its parental MOS cell line (Table 1, Figure 1C). Moreover, 1 *μ*M verapamil augmented the effects of ZOL on MOS/ADR cells and restored the sensitivity of MOS/ADR cells to almost same level of its parental cell line, MOS. These findings suggested that the effects of ZOL in osteosarcoma cells were affected by P-gp. However, we have previously reported that the antitumour effects of ZOL against leukaemic cell lines are not affected by P-gp (Kuroda *et al*, 2003). This discrepancy could be explained by the differences on the activity of ZOL against leukaemic cells and osteosarcoma cells. Both IC_50_s of ZOL against parental and P-gp-overexpressing leukaemic cells are above 60 *μ*M, whereas of those against osteosarcoma cells were much less. It could, therefore, be argued that in the cell lines that we used, the efflux pump enabled the elimination of small amounts of BPs, although it did not work efficiently for large quantities of ZOL.

According to a previous study evaluating ZOL efficacy for the treatment of osteoporosis (Chen *et al*, 2002), peak serum concentrations were in the range of 1–3 *μ*M and maintained for only a few hours. As IC_50_s of ZOL for MOS and LM8 cells are 1.56 and 7.36 *μ*M, respectively, it is likely that the effect of ZOL alone might be insufficient. Moreover, ZOL is less effective against P-gp-overexpressing osteosarcoma cells. Therefore, we examined the combined effects of ZOL with other anticancer agents. In the present study, we examine the combined effects of ZOL with commonly used anti-osteosarcoma agents such as DOX, CDDP and MTX. CDDP has been reported to augment synergistically the effects of ZOL (Matsumoto *et al*, 2005). We also examine the combined effects of ZOL with other three agents that are undergoing clinical trial or have been examined *in vitro* experiments for osteosarcoma cells, such as IM (McGary *et al*, 2002), PAC (Verweij *et al*, 2000) and GEM (Okuno *et al*, 2002). IM specifically inhibits selected tyrosine kinase receptors, including platelet-derived growth factor (PDGF) and c-Kit. As osteosarcoma expresses low levels of c-Kit, but abundant levels of the PDGF receptor (McGary *et al*, 2002), IM might be a promising candidate for osteosarcoma therapeutics. Both PAC, a mitotic spindle toxin and GEM, a nucleoside analogue, are in phase 2 clinical trails for osteosarcoma (Verweij *et al*, 2000; Okuno *et al*, 2002).

The concurrent treatment of ZOL with DOX or CDDP resulted in additive or synergistic growth inhibition of osteosarcoma cell lines. Interestingly, concurrent exposure to ZOL significantly augmented the effects of PAC and GEM in these cell lines. When MOS or LM8 cells were treated with ZOL combined with PAC for 24 h, there was an increase in the proportion of MOS cells in S-phase despite the fact that neither drug at this concentration affected the cell cycle when used individually (Figure 5A, B-1-4). These suggested that when ZOL is combined with PAC, PAC might augment the ability of ZOL to produce S-phase arrest, resulting the combined effects. Similarly, when LM8 cells were treated with ZOL combined with GEM at the concentration which had no effects on the alteration on cell cycle for 24 h, there was a significant increase in the ratio of cells in sub-G_1_ (Figures 5B-2, 5 and 6). This indicated that when combined with GEM, ZOL might induce more apoptotic cells. These synergistic effects of ZOL depend on the doses and the osteosarcoma cell line studied.

These results may have therapeutic application, particularly for enhancing the efficacy of DOX or CDDP that cannot be administered at higher dosages because of toxicity. Recently, the combined effects of ZOL and PAC (Jagdev *et al*, 2001; Neville-Webbe *et al*, 2006) or GEM (Budman and Calabro, 2006) have been reported. However, the precise mechanism by which ZOL enhances the effects of these agents is not yet fully understood. We tried to investigate the mechanism of combined effects with ZOL and other agents. As we have previously reported in leukaemic cells, ZOL inhibited the progression of osteosarcoma cells in S-phase (Table 3). Two agents that showed significant combined effects with ZOL such as PAC and GEM revealed different effects of the alteration of cell cycle in osteosarcoma cells. PAC accumulated cells in G_2_/M phase, resulting the induction of apoptosis (Das *et al*, 2001), while GEM seemed to induce apoptosis in S phase (Shi *et al*, 2001) (Table 3). These findings suggest that it is difficult to predict which agent becomes a good partner for ZOL based on its activity on the alteration of cell cycle. Further studies will be required to more fully elucidate these mechanisms.

P-gp-mediated multi-drug resistance is crucial for cancer treatment. Although many P-gp inhibitors have been identified, none of them have been proven clinically useful without side effects, combination chemotherapy is one of the strategies to overcome the P-gp mediated multi-drug resistance (Ozben, 2006). Based on the additional effect of verapamil on the ZOL- or DOX-induced growth inhibition of MOS/ADR cells (Figure 1B and C), P-gp was suggested to have some role in ZOL-resistance. Because PAC is a substrate for P-gp (Horwitz *et al*, 1993) (Table 1), it is suggested that the coadministration of PAC and ZOL show the inhibitory effect on the cell growth of MOS/ADR cells by similar mechanism with the addition of verapamil. In the present study, we have found the four-fold resistance against GEM in the MOS/ADR cells in comparison with the parental cells (Table 1). However, the additional effect of GEM on the ZOL-induced inhibitory effect on cell growth was observed both in the parental MOS and MOS/ADR cells (Figure 2B and C). Therefore, the coadministration of ZOL may independently inhibit the cell growth of these cell lines in addition to GEM, which shows its effects via deoxycytidine kinase pathway (Blackstock *et al*, 2001). The mechanism of these combined effects should be more investigated. However, ZOL plus PAC or GEM might become a good application for multi-drug-resistant osteosarcoma cells.

We also investigated the sequential combined effects of ZOL with other anticancer agents. MOS and LM8 cells were pretreated for 24 h with lower concentration of ZOL, and that concentration did not alter the cell cycle and increase the percentage of apoptotic cells in these cells, and significantly augmented the effects of DOX in both MOS and LM8 cells (Figure 3A and 4A), and of CDDP in LM8 cells (Figure 4B). In contrast, ZOL combined with MTX in both cell lines showed antagonistic effects (Figure 3C and 4C). This antagonistic effect of MTX was also seen in leukaemic cells (Kimura *et al*, 2004). Because MTX activity was changed by the sensitivity of cells and the pharmacokinetics of the drugs, MTX has major cytotoxic effects on cells in the S phase and accumulates in cells in the G_1_ to early S phase and later into the G_2_/M phase (Lorico *et al*, 1990; Yamauchi *et al*, 2005). On the contrary, ZOL has major cytotoxic effects in the S phase and accumulates in cells in the late S and early G_2_ phases (Kuroda *et al*, 2003). If cells were treated MTX followed by ZOL, the combined effect may be observed to augment each other. But in the present study, the combined treatments of ZOL with MTX have been carried out. It has been noted that one agent might reduce the cytotoxicity of the other agent by preventing cells from entering the specific phase in which the cells are most sensitive to the other agent. Although the reason for this antagonism is not fully clarified, the simultaneous administration of ZOL with MTX might be counterproductive not only for treatment of osteosarcoma but also for other cancers. Methotrexate is commonly used in various chemotherapy regimens (Kummel *et al*, 2006). Therefore, the clinical use of ZOL should be evaluated carefully to avoid inadequate interaction even when ZOL is indicated for other reasons, such as metastatic involvement of the bone.

In conclusion, the combination of ZOL with DOX, CDDP, PAC or GEM may be effective against murine osteosarcoma cells, compared to the use of any of these agents alone. These results provide a basis for conducting further studies using human osteosarcoma cell lines or fresh osteosarcoma cells obtained from patient resection samples and such studies ultimately will provide a rationale for the preclinical/clinical evaluation of the antitumour activity of ZOL in combination with other anticancer agents.

## Figures and Tables

**Table 1:** The IC_50_^a^ values (*μ*M) of ZOL and anticancer agents in murine osteosarcoma cell lines

**Agents**	**YM529**	**ZOL**	**DOX**	**CDDP**	**MTX**	**IM**	**PAC**	**GEM**
LM8	6.20	7.36	0.30	11.0	0.12	5.6	0.025	0.32
MOS	1.22	1.56	0.19	16.9	0.039	15.0	0.048	0.21
MOS/ADR	5.90	7.10	0.82	—	—	—	0.16	0.89
Resistance^b^	4.8	4.6	4.4	—	—	—	3.3	4.2

Values represent the means of at least three independent experiments.

a IC_50_=the drug concentration yielding 50% growth inhibition.

b The level of resistance to each agent is expressed as the IC_50_ of MOS/ADR cells divided by the value of MOS cells.

YM529 data have been reported elsewhere (Horie *et al*, 2006).

**Table 2:** Combination indexes at Fa 0.50 and 0.80 of ZOL in concurrent combination with other agents

**Agents**	**Cell line**	**CI at Fa 0.5 (Effect)**	**CI at Fa 0.8 (Effect)**
DOX	MOS	1.14±0.09 (Antagonism)	0.97±0.17 (Additive)
	LM8	1.01±0.14 (Additive)	0.50±0.19 (Synergism)
CDDP	MOS	0.35±0.06 (Synergism)	0.59±0.14 (Synergism)
	LM8	0.83±0.24 (Additive)	0.48±0.13 (Synergism)
MTX	MOS	1.52±0.73 (Additive)	1.15±0.92 (Additive)
	LM8	0.97±0.17 (Additive)	0.93±0.31 (Additive)
IM	MOS	0.14±0.11 (Synergism)	0.72±0.65 (Additive)
	LM8	1.06±0.19 (Additive)	1.24±0.39 (Additive)
PAC	MOS	0.07±0.05 (Synergism)	0.21±0.27 (Synergism)
	LM8	0.03±0.07 (Synergism)	0.52±0.21 (Synergism)
GEM	MOS	0.18±0.10 (Synergism)	0.26±0.31 (Synergism)
	LM8	0.67±0.12 (Synergism)	0.53±0.22 (Synergism)

**Table 3:** Summary of cell cycle distribution (%) following 24 h agent treatment in osteosarcoma cells (A: MOS cells, B: LM8 cells)

	**Sub-G_1_**	**G_1_**	**S**	**G_2_/M**
A-1: untreated MOS cells	3.7	46.2	30.8	16.8
A-2: ZOL (2.0 *μ*M)	5.5	32.1	46.3	12.6
A-3: PAC (25 nM)	17.5	19.7	18.8	28.9
B-1: untreated LM8 cells	4.7	46.2	25.0	21.4
B-2: ZOL (15 *μ*M)	6.6	30.0	39.0	21.0
B-3: PAC (10 nM)	27.0	2.4	10.2	47.0
B-4: GEM (25 nM)	25.1	30.7	25.6	10.4
